# Investigating the Risk Indicators of Urinary Incontinence Among Young Nulligravid Women: A Cross-Sectional Study

**DOI:** 10.1089/whr.2025.0004

**Published:** 2025-05-12

**Authors:** Ghada Mohammed, Noha A. Mousa, Shaikha S. Alhaj, Basema Saddik

**Affiliations:** ^1^ Clinical Sciences Department, College of Medicine, University of Sharjah, Sharjah, United Arab Emirates.; ^2^ Department of Family and Community Medicine and Behavioral Sciences, College of Medicine, University of Sharjah, United Arab Emirates.; ^3^ Sharjah Institute of Medical and Health Sciences, University of Sharjah, United Arab Emirates.; ^4^ School of Population Health, Faculty of Medicine and Health, University of New South Wales, Australia.

**Keywords:** urinary incontinence, stress incontinence, urge incontinence, toilet behavior, nulligravida

## Abstract

**Background:**

Urinary incontinence (UI) and associated lower urinary tract symptoms (LUTS) are well documented in older, multiparous women, with established risk factors such as menopause, neurological disorders, and diabetes mellitus. However, emerging evidence indicates that young, nulligravid women without these traditional risk factors may also be affected. This study explores the prevalence, risk factors, and impact of UI and LUTS in this population.

**Methods:**

A cross-sectional study was conducted using an anonymous online questionnaire adapted from the International Consultation on Incontinence Questionnaire for Female Lower Urinary Tract Symptoms and the Lower Urinary Tract Symptoms Quality of Life. Participants were women aged 18–25 years who had never been pregnant.

**Results:**

Approximately one-third of participants reported experiencing UI (urge, stress, or mixed incontinence), whereas 45.9% reported at least one LUTS without UI. Significant associations were identified between UI and increased body mass index (*p* = 0.007), smoking (*p* = 0.018), and recurrent urinary tract infection (*p* = 0.004). Toilet behaviors, such as delaying urination until bladder fullness, were also significantly associated with UI. Logistic regression analysis identified key predictive risk factors for UI: being overweight or obese (odds ratio [OR] = 1.88, confidence interval [CI] = 1.22–2.90), smoking (OR = 3.07, CI = 1.32–7.12), and delaying bladder emptying (OR = 2.99, CI = 1.63–5.47). Women with UI self-reported significant bother from symptoms, particularly those with overactive bladder (urge incontinence: 72.3%, urinary urgency: 53.6%, and nocturia: 55.4%). Quality of life was notably impacted, with 28.3% of participants with urge incontinence requiring daily pad use. Despite this, the majority (85.1%) did not seek medical care.

**Conclusions:**

UI and LUTS are prevalent in young nulligravid women, with modifiable risk factors such as lifestyle habits and toilet behaviors playing a critical role. These findings highlight the need for community awareness programs and proactive patient education during clinical encounters, as affected women are unlikely to seek medical advice voluntarily.

## Introduction

Bladder dysfunction is defined as the presence of bothersome lower urinary tract symptoms (LUTS), including urinary incontinence (UI).^1^ UI is a common and distressing condition that is often underreported in women. According to the International Continence Society, UI is defined as the “involuntary loss of urine that is objectively demonstrable.”^2^ Although UI is not life-threatening, it can negatively impact women’s psychological well-being and interfere with various aspects of their daily lives and social activities, ultimately diminishing their overall quality of life. Studies have demonstrated its influence on areas such as relationships, employment, travel, sports, and leisure activities.^3^ Moreover, there is a higher prevalence of major depression among women dealing with incontinence.^4^ In addition, despite the reported prevalence of some degree of UI in 15%–50% of women, only one in four women seek medical help.^5,6^ Reasons cited for not seeking help include embarrassment or considering the condition a normal consequence of childbirth or aging. Women often have low expectations of treatment or believe that treatments are unavailable.^7^

The prevalence and risk factors of UI in parous and older women have been extensively studied across different populations, identifying pregnancy and childbirth as significant contributors due to hormonal changes, mechanical stress on the pelvic floor, and potential nerve injury during labor.^8–12^ UI and its predisposing conditions in young women who have never been pregnant remain understudied. A study found the prevalence of UI in nulligravid women aged 16–30 years ranged between 12.6% and 14.7%.^13^ Moreover, in a study of healthy nulligravid women aged 18–30 years, 94.3% of the participants reported LUTS, whereas 20.1% reported having UI.^14^ A systematic review examined the prevalence of UI in nulliparous adolescent and middle-aged women and reported a prevalence of UI ranging between 10.8% and 42%.^15^ Studies included in this systematic review identified several risk factors for developing UI in this cohort. Age, body mass index (BMI), childhood enuresis, anxiety, psychological disorders, eating disorders, constipation, sexual activity, and hormonal contraception are among the reported risk factors.^15^ Additionally, toileting behaviors were significantly found to be related to LUTS and the development of UI.^16–18^

Although numerous studies in the Gulf region have examined the risk factors and impact of UI and LUTS in women, research specifically focusing on young nulligravid women remains limited.^11^ Understanding the prevalence and risk factors of UI in this population is crucial for developing targeted prevention and intervention strategies. Identifying modifiable risk factors and implementing appropriate measures may help mitigate the burden of UI and enhance overall well-being. This study aims to determine the prevalence of UI among young nulligravid women aged 18–25 years, investigate modifiable risk factors associated with its development, and evaluate its impact on daily functioning, mental health, and quality of life.

## Materials and Methods

This cross-sectional study includes nulligravid women aged 18–25 years old. The study has obtained ethical approval from the Research Ethics Committee at the University of Sharjah in the United Arab Emirates (Reference Number: REC-23-03-29-03-F). The invitation to participate in the study was sent through different social media platforms. The invited participants were provided with an information sheet stating the purpose of the study (participant information sheet is available as supplemental material). Anonymized data were collected from participants after obtaining informed consent. The questionnaire was administered using Microsoft Forms, in both English and Arabic to match the diversity of the study population. Utilizing a previously reported UI prevalence of 42% among the United Arab Emirates’ women,^12^ the minimum required sample size was determined to be 375 participants. This calculation was performed using the OpenEpi Sample Size Calculator, with a 95% confidence interval (CI), a margin of error of 5%, and a power of 80%. A 20% augmentation was incorporated to accommodate potential nonresponses and incomplete surveys, resulting in a final minimum required sample size of 450 participants. To mitigate the risk of missing data and enhance generalizability, 635 participants were ultimately included in the study.

The study targeted healthy women aged 18–25 who were neither pregnant nor had ever been pregnant, excluding those with diabetes mellitus or any urological or neurological conditions. The participants were selected through convenience sampling, and the questionnaire was distributed *via* social media platforms. A validated questionnaire comprising 49 items, adapted from the International Consultation on Incontinence Questionnaire for Female Lower Urinary Tract Symptoms (ICIQ-FLUTS)^19^ and Lower Urinary Tract Symptoms Quality of Life (ICIQ-LUTSqol),^20^ was employed for data collection. The questionnaire was organized into four sections:

i) Demographics and medical history: Included variables such as age, education level, marital status, ethnic group, and history of medical conditions such as urinary tract or bladder infections, lung diseases, and depression.
ii) Assessment of risk factors: Evaluated risk factors such as height, weight, frequency and type of exercise, history of smoking or diuretic use, and caffeine intake.
iii) Bladder symptoms: Examined urination habits, including the frequency of bladder emptying during the day and night, incidence of pain during urination, and occurrence of urine leakage during exercise or physical activity.
iv) Impact on the quality of life: Assessed the repercussions of these symptoms on the respondents’ quality of life.

The questionnaire responses were anonymized and stored on a secured computer in an encrypted folder protected with a password to ensure confidentiality. Both English and Arabic versions of the questionnaire were utilized for data collection. The questionnaire was originally developed in English and translated into Arabic by a trained translator. To ensure the accuracy of the translation, the Arabic version was back-translated into English. This back-translation process helped identify and rectify any discrepancies, ensuring that both versions were equivalent in meaning,

To ensure face and content validity, the final version of the questionnaire in both languages was reviewed by a panel of experts in the field. These experts provided feedback on the clarity, relevance, and comprehensiveness of the questionnaire items. Additionally, a pilot test of the questionnaire was conducted with a sample of 10 women who were fluent in either English or Arabic. The pilot study aimed to identify any areas of ambiguity or confusion in the questions. Based on the feedback from the pilot study, necessary modifications were made to the questionnaire to enhance its clarity and comprehensibility.

Subsequently, the collected data were coded and analyzed using IBM Statistical Package for Social Sciences for Windows, Version 21 (IBM Corp, Armonk, NY, USA). Categorical variables were expressed as frequencies (n) and percentages (%), and differences between groups were compared using chi-square tests. Multivariate logistic regression was performed to identify which factors remained independently associated with UI after adjusting for potential confounders. The logistic regression model included variables that were found to be significant in the bivariate analysis or were theoretically relevant based on existing literature. Results for the regression are expressed in adjusted odds ratios (ORs) with 95% CIs. The significance level was set at *p* < 0.05.

## Results

The total number of women who participated in this study was 635. All participants were 25 years of age or younger and had never been pregnant. Only 4.4% of the participants were married. The majority (82.5%) were undergraduates (university students). Notably, over one-third of the study participants were overweight or obese. The majority were nonsmokers with no significant medical history (Table 1).

**Table 1.** Demographics, Lifestyle, Clinical Characteristics, and Toilet Behavior of the Study Population (*n* = 635)

	*n*	%		*n*	%
Demographics		* *	Toilet behavior	* *	* *
Age group			Empty bladder even if does not need to		
18–20	353	55.59	Never	110	17.32
21–25	282	44.41	Rarely	210	33.07
Education degree			Sometimes	271	42.68
Secondary school	57	8.98	Often/always	44	6.93
Undergraduate	524	82.52	Delay going to the toilet until full bladder		
Postgraduate	54	8.5	Never	42	6.61
Marital status			Rarely	142	22.36
Single	607	95.59	Sometimes	308	48.5
Married	28	4.41	Often/Always	143	22.52
Ethnicity			Avoid using public toilets		
Arab	509	80.16	No	167	26.3
African	30	4.72	Yes	468	73.7
Others	96	15.12	Medical history	* *	* *
BMI			Diagnosed with urinary tract or bladder infections more than 3 times in a year		
Underweight	331	56.01	No	549	92.58
Normal	78	13.2	Yes	44	7.42
Overweight	120	20.3	Ever diagnosed with depression		
Obesity	62	10.49	No	473	85.38
Lifestyle	* *	* *	Yes	81	14.62
Exercise			Ever diagnosed with lung disease/asthma		
No	362	57.01	No	552	90.49
Yes	273	42.99	Yes	58	9.51
Frequency of exercise					
Not regular	85	31.14			
1–3 times per week	115	42.12			
More than 3 times per week	73	26.74			
Taking diuretics or “water pills”					
No	625	98.43			
Yes	10	1.57			
Currently smoke					
No	598	94.17			
Yes	37	5.83			
Ever been lifting more than 20 pounds regularly					
No	391	78.99			
Yes	104	21.01			
Drink more than one cup of tea/coke/coffee					
No	334	52.6			
Yes	301	47.4			
Total	635	100			

BMI, body mass index.

**FIG. 1.**
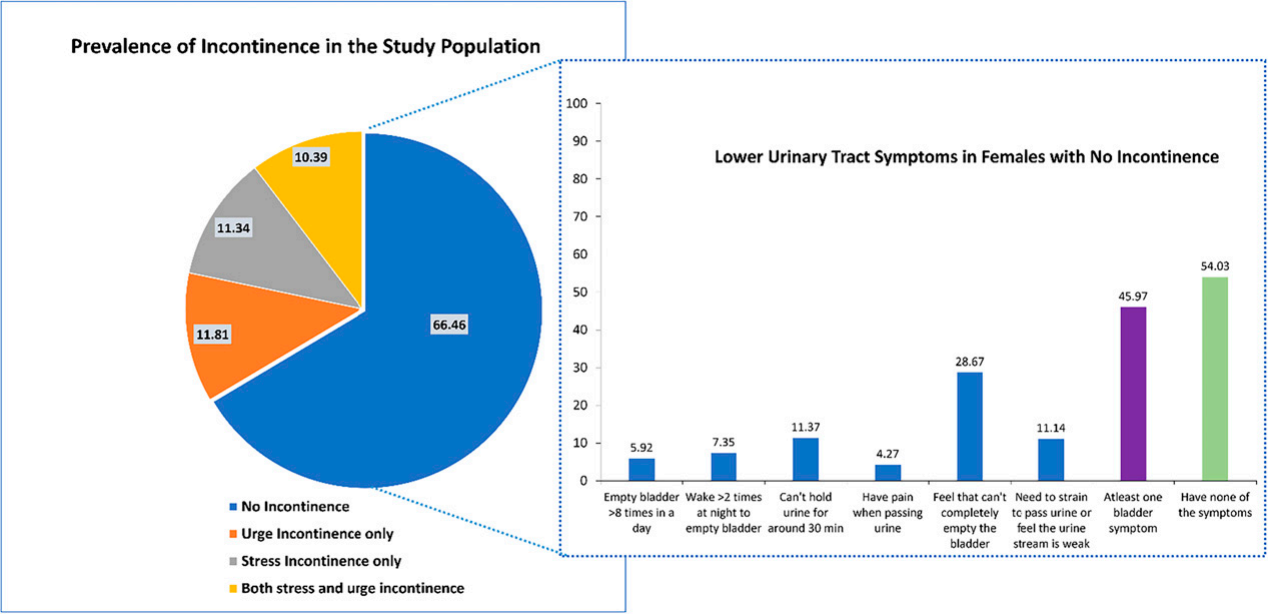
The prevalence of the different types of UI and the prevalence of different LUTS among participants without UI. LUTS, lower urinary tract symptom; UI, urinary incontinence.

The study identified that approximately one-third (33.5%) of the participants (*n* = 213) reported having UI (including stress, urge, and mixed incontinence). Moreover, 45.9% of participants who do not have UI (*n* = 422) reported at least one of the LUTS (Fig. 1).

Various potential clinical and lifestyle factors linked to an increased risk of UI were investigated among the study population. A statistically significant association was identified between UI and increased BMI, smoking, and a history of recurrent urinary tract infection (UTI). Regarding toilet behavior, significant associations were found between UI and both emptying the bladder without the need to and delaying bladder emptying until it is full (Table 2).

**Table 2.** Associations Between Demographic, Medical, and Behavioral Risk Factors, and Urinary Incontinence in the Study Population

	*n*	%	*p*
Demographic characteristics			
Age group			
18–20	116	32.9	0.0684
21–25	97	34.4	
Marital status			
Single	201	33.1	
Married	12	42.9	0.286
BMI			
Underweight/normal	124	30.32	
Overweight/obese	76	41.76	**0.007**
Behavioral factors			
Exercise			
No	129	35.6	
Yes	84	30.8	0.199
Frequency of exercise			
Not regular	24	28.2	
1–3 times per week	42	36.5	
More than 3 times per week	18	24.7	0.19
Currently smoke			
No	194	32.4	
Yes	19	51.4	**0.018**
Drink more than one cup of tea/coke/coffee			
No	102	30.5	
Yes	111	36.9	0.091
Medical history			
Diagnosed with urinary tract or bladder infections more than 3 times in a year			
No	170	31	
Yes	23	52.3	**0.004**
Ever diagnosed with depression			
No	148	31.3	
Yes	33	40.7	0.094
Ever diagnosed with lung disease/asthma			
No	177	32.1	
Yes	24	41.4	
Toilet behaviors	213	33.5	0.151
Empty bladder even if do not need to			
Never/rarely	94	29.4	
Sometimes	99	36.5	
Often/always	20	45.5	**0.041**
Delay going to the toilet until full bladder			
Never/rarely	45	24.5	
Sometimes	103	33.4	
Often/always	65	45.5	**0.001**
Avoid using public toilets			
No	58	34.7	
Yes	155	33.1	0.705

The bold data are the ones that are statistically significant based on the p value and the odd ratio.

Logistic regression analysis identified potential predictive risk factors for the development of UI in the study population. Women with an increased BMI (≥25) had a significantly higher risk of developing any type of UI compared with women with normal or low BMI. Study participants who smoked had a three times higher risk compared with nonsmokers. Likewise, women who delayed emptying their bladder until feeling full were found to have almost three times significantly higher risk of developing UI (Table 3).

**Table 3.** Logistic Regression Analysis for Women Reporting Urinary Incontinence and Demographic, Behavioral, and Medical Factors

	Odds ratio	95% CI	*p*
Marital status			
Single	1		
Married	1.71	(0.71–4.10)	0.23
BMI			
Underweight/normal	1		
Overweight/obese	1.88*	(1.22–2.90)	**0.001**
Exercise			
No	1		
Yes	0.89	(0.59–1.35)	0.58
Currently smoke			
No	1		
Yes	3.07*	(1.32–7.12)	0.01
Drink more than one cup of tea/coke/coffee			
No	1		
Yes	1.33	(0.88–2.01)	0.17
Toilet behaviors Empty bladder even if do not need to			
Never/rarely	1		
Sometimes	1.31	(0.85–2.02)	0.22
Often/always	1.63	(0.76–3.49)	0.21
Delay going to the toilet until the bladder is full			
Never/rarely	1		
Sometimes	1.57	(0.95–2.60)	0.08
Often/always	2.99*	(1.63–5.47)	**0.001**
Avoid using public toilets			
No	1		
Yes	0.73	(0.45–1.17)	0.19
Medical history			
Diagnosed with urinary tract or bladder infections more than 3 times in a year			
No	1		
Yes	1.21	(0.53–2.78)	0.65
Ever diagnosed with depression			
No	1		
Yes	1.06	(0.58–1.96)	0.85
Ever diagnosed with lung disease/asthma			
No	1		
Yes	1.39	(0.71–2.70)	0.33

The bold data are the ones that are statistically significant based on the p value and the odd ratio.

CI, confidence interval; OR, odds ratio.

**FIG. 2.**
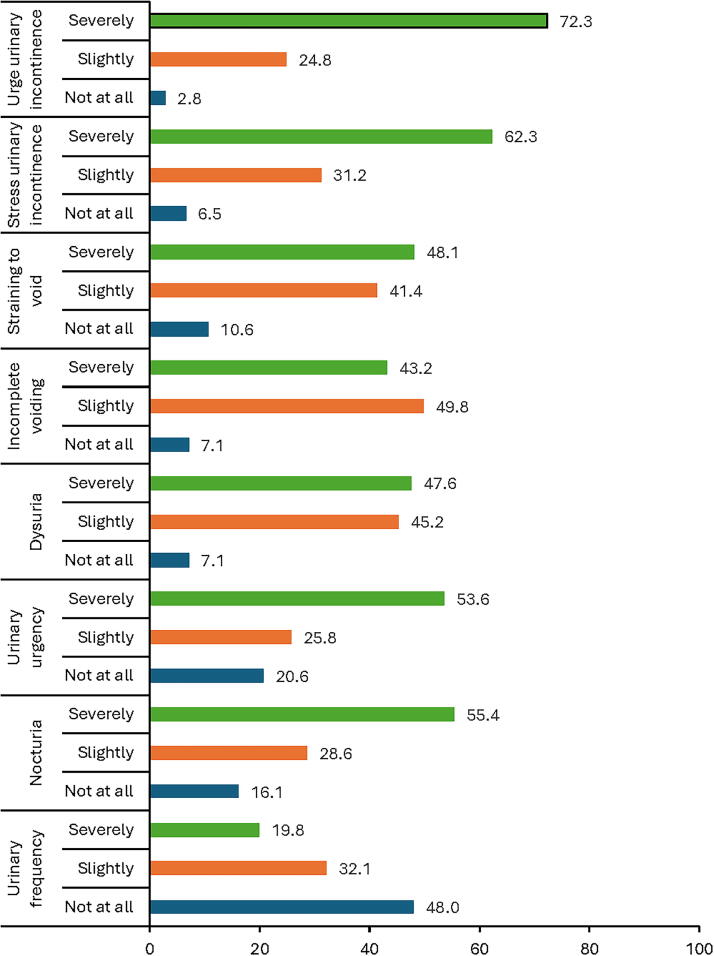
Percentages of the level of bothersomeness from LUTS and UI among the study participants (the numbers of participants in each category are available as Supplemental Fig. S1).

The level of impact of LUTS and UI on the study participants was evaluated. Most women who reported UI, whether urge or stress incontinence, were severely affected by their symptoms (72.3%/*n* = 102 and 62.3%/*n* = 86, respectively). Among the women affected by LUTS, the highest impact was reported by those who suffered from nocturia (55.4%/*n* = 31) and urinary urgency (53.6%/*n* = 52) (Fig. 2 and Supplementary Fig. S1).

**FIG. 3.**
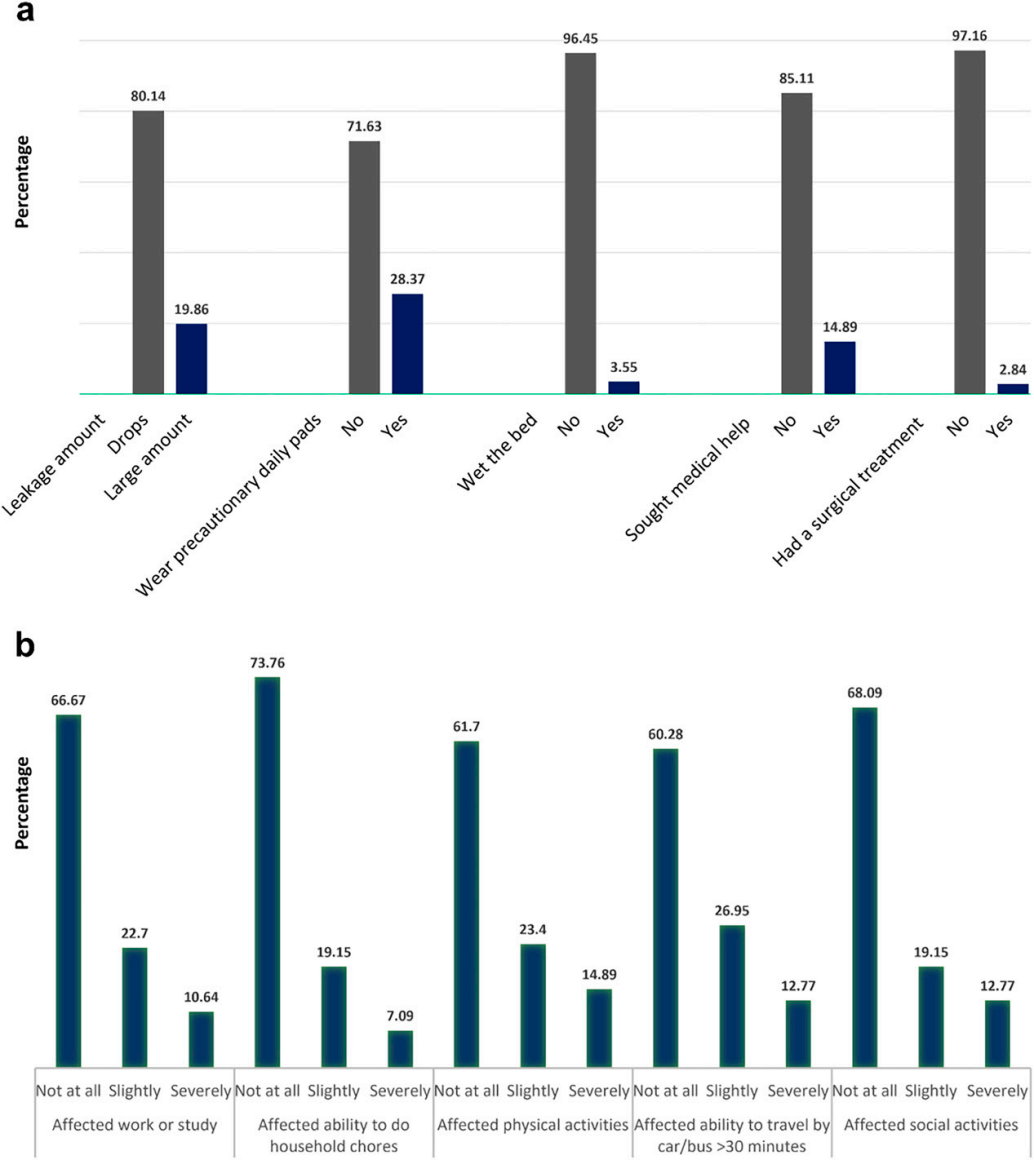
QOL assessment in a subgroup of women with UUI demonstrates (a) indicators of the severity of symptoms and (b) the impact on daily physical and social activities.

Women with urge UI (*n* = 141) were further evaluated for the effect on quality of life including several objective indicators of the severity of their symptoms and the extent of the impact on their daily physical and social activities. Although almost one-third (28.3%/*n* = 40) of the affected women needed to wear daily pads, the majority (85.1%/*n* = 120) did not seek medical help (Fig. 3a and b).

## Discussion

This study found the prevalence of UI to be surprisingly high in a cohort of young nulligravid women (33.5%) compared with previously published estimates ranging between 12% and 20%.^13,14,21^ UI prevalence typically increases with age. In a large Norwegian population study of around 28,000 participants, UI was least prevalent among younger women under 30 (12%) and highest among the oldest groups (40%). There was also a noticeable increase in prevalence around middle age (30%), followed by a steady rise in the elderly, reaching up to 50%.^22^ A recent systematic review reported UI prevalence among women in the Gulf region to range between 20.3% and 54.5%.^11^ Among women attending primary health care centers in Saudi Arabia, the prevalence of UI was 41.4%.^10^ Similarly, among women in the United Arab Emirates, the prevalence of UI was 42%.^12^ Research on the prevalence of UI among young nulligravid women, particularly in the Middle East, is scarce. Most existing studies have focused on the general female population rather than specifically addressing nulligravid women. Moreover, our findings revealed that a considerable proportion (45.9%) of young nulligravid women experience LUTS without UI, highlighting the broader spectrum of bladder health issues in this group.

Approximately one-third (30.8%) of the participants were classified as overweight or obese, which is consistent with the findings from epidemiological studies on obesity prevalence in this young age group of women.^23^ Obesity has been identified as a risk factor for developing UI in several studies.^24^ Our study found a significant association between increased BMI and UI in young women (*p* = 0.007). Specifically, obesity and overweight were significant predictors of UI risk (OR = 1.88) in this group. This finding is concerning given the global rise in childhood and adolescent obesity.^25^ Similarly, smoking was significantly associated with UI in our study, in line with previously published data.^26,27^ However, only 5.8% of our participants reported smoking, which is comparable with the global prevalence of smoking among women as reported by the World Health Organization (7.9% in 2020, projected to decline to 6.7% by 2025).^28^

In our study, 7.4% of women reported recurrent episodes of UTI (defined as more than three times per year). There was a significant association between recurrent UTI and UI (*p* = 0.004). Acute UTIs are often accompanied by transient bladder symptoms such as urgency, dysuria, frequency, and even UI.^29^ Recurrent UTI may lead to chronic subclinical bladder infections, predisposing affected women to the development of overactive bladder symptoms.^30^ Although the underlying mechanism remains unclear, it could involve persistent irritation of the bladder mucosa, nerve endings, and or muscle tone of the bladder. Recent evidence also indicates that changes in the bladder microbiota may contribute to the development of bladder overactivity and incontinence.^31^

The impact of toilet behavior on bladder function has been well recognized as a risk factor for developing LUTS and UI in women of all ages, including young women.^18,32,33^ In our study, women who often or always delayed emptying their bladder had a significant threefold increased risk of developing UI (OR = 2.99, *p* = 0.001). This might be partly related to the common practice of avoiding public toilets, which was reported by 72.7% of those with UI symptoms. Habitual convenience voiding (emptying the bladder without the need to) was also significantly associated with UI (*p* = 0.04), aligning with findings from similar studies conducted in comparable age groups.^34–36^

Among our participants, urinary leakage was reported as the most bothersome symptom, including both stress and urge UI (UUI). Other LUTS, particularly overactive bladder symptoms such as nocturia and urgency, were also reported as bothersome. These findings align with previously published studies.^37,38^

Women with UUI in our study were further evaluated for symptom severity and its impact on their quality of life. Between 7% and 14% reported significant effects on their work, studies, physical and social activities, or daily household tasks. Additionally, nearly one-third of these women indicated they used two or more pads daily to prevent urinary leakage. These findings are concerning, particularly given the typically active lifestyles of this young age group. Similar findings on the negative effect on quality of life were reported in a previously published study.^39^

Despite the significant impact on quality of life, the majority (85.1%) of women in our study did not seek medical help. Although reasons for not seeking medical help were not explored in this study, previous research has identified embarrassment and a lack of awareness of where to seek help as common barriers.^40,41^ It is reasonable to assume that these factors may be even more pronounced among younger women from diverse cultures in our study. This emphasizes the importance of community awareness about bladder health and toileting behaviors. Further studies focusing on this group of young women are recommended to explore other risk factors as well as their perceptions of LUTS and UI.

One limitation of our study is the reliance on self-reported data, which may introduce recall bias or inaccuracies in participants’ responses. BMI was calculated from the reported height and weight of the participants through the online questionnaire (93% of the participants responded to the question). Additionally, the cross-sectional design and the use of convenience sampling limit the ability to establish causal relationships between risk factors and UI and may not allow the generalizability of results. However, despite these limitations, the findings from this study should not be overlooked as they provide important baseline data on UI in young, nulligravid women, which will be valuable in guiding future research.

## Conclusions

UI and LUTS are prevalent among young, nulligravid women, with significant associations identified between UI and modifiable risk factors such as elevated BMI, smoking, and delayed bladder emptying. The findings suggest that lifestyle factors and toilet behaviors contribute to the development of UI in this population. Despite the considerable impact on quality of life, most affected women do not pursue medical care. These results emphasize the need for targeted public health interventions and proactive clinical education to raise awareness and improve the management of UI in young women.

## Abbreviations Used

BMI: Body Mass Index
CI: Confidence Interval
ICIQ-FLUTS: International Consultation on Incontinence Questionnaire for Female Lower Urinary Tract Symptoms
ICIQ-LUTSqol: International Consultation on Incontinence Questionnaire for Lower Urinary Tract Symptoms Quality of Life
ICS: International Continence Society
LUTS: Lower Urinary Tract Symptoms
OR: Odd Ratio
UI: Urinary Incontinence
UTI: Urinary Tract Infection
UUI: Urge Urinary Incontinence
