# Supplementary material for: Investigating the Risk Indicators of Urinary Incontinence Among Young Nulligravid Women: A Cross-Sectional Study
Source: Womens Health Rep (New Rochelle). 2025 May 12;6(1):546–55. doi: 10.1089/whr.2025.0004 (PMC12177332; doi:10.1089/whr.2025.0004)
Supplement: Supplementary Data S1 [file whr.2025.0004_supplementary_data_s1.pdf]

جامعـــــة الشـــــارقة
UNIVERSITY OF SHARJAH

Determining the Risk of Urinary Incontinence among Young Nulligravid Women Questionnaire

Greetings,

We are a research team from the College of Medicine, University of Sharjah. We are inviting you to participate in a study by taking a short survey. The purpose of this study is to assess how common is urinary leakage among young women who never got pregnant in the UAE female population. We also try to understand which factors may lead to this problem to help increase awareness and provide proper preventive measures.

You are invited to participate if you meet the following criteria:

1. Your age is between 18-25 years.
2. You are single, married, or have been married before.
3. You are not currently pregnant and have never been pregnant before.
4. You do not have Diabetes Mellitus, neurological disease, or urological disease.

Participation is voluntary and anonymous.

You have the right to withdraw from this study at any time.

Agreeing to fill out this survey is considered your informed consent. The survey will take around 5 minutes.

There are no risks associated with participation in this study. The questionnaire is anonymous, and we assure you that responses will be confidential and will be used only for research purposes.

For any feedback or questions please contact:
Dr. Ghada Mohammed, Email: gmohammed@sharjah.ac.ae
Telephone number: 065057209/0506289322

In case of concerns or complaints regarding your participation in this research, you may contact Dr. Suhail Al Amad, the head of the research ethics committee at: 06-5057304 or by Email: REC@sharjah.ac.ae

تحديد خطر سلس البول بين النساء الشابات اللواتي لم يسبق لهن الحمل
استبانة

نحن فريق بحثي من كلية الطب ، جامعة الشارقة. ندعوك للمشاركة في دراسة من خلال إجراء استطلاع قصير. الغرض من هذه الدراسة هو تقييم مدى شيوع مشكلة تسرب البول بين الشابات اللواتي لم يحملن مطلقًا بين الإناث في الإمارات العربية المتحدة. نحاول أيضًا فهم العوامل التي قد تؤدي إلى هذه المشكلة للمساعدة في زيادة الوعي وتوفير التدابير الوقائية المناسبة.

أنت مدعوة للمشاركة إذا كنت تستوفين المعايير التالية:

1. عمرك بين 18-25 سنة.
2. أنت عزباء أو متزوجة أو كنت متزوجة من قبل.
3. أنتِ لستِ حامل حاليًا ولم تحملي من قبل.
4. ليس لديك مرض السكري أو مرض عصبي أو أمراض المسالك البولية.

المشاركة طوعية ومجهولة.

لديك الحق في الانسحاب من هذه الدراسة في أي وقت.

الموافقة على ملء هذا الاستبيان تعتبر موافقتك المستنيرة. سيستغرق الاستبيان حوالي 5 دقائق.

لا توجد مخاطر مرتبطة بالمشاركة في هذه الدراسة. الاستبيان مجهول الهوية ، ونؤكد لك أن الردود ستكون سرية ولن تستخدم إلا لأغراض البحث.

لأية ملاحظات أو أسئلة ، يرجى الاتصال بـ:

د.غادة محمد ، بريد الكتروني: gmohammed@sharjah.ac.ae
رقم الهاتف: 0506289322/065057209

في حالة وجود مخاوف أو شكاوى بخصوص مشاركتك في هذا البحث ، يمكنك الاتصال بالدكتور سهيل العمد ، رئيس لجنة أخلاقيات البحث على: 06-5057304 أو عن طريق البريد الإلكتروني: REC@sharjah.ac.ae
