# Supplementary material for: Investigating the Risk Indicators of Urinary Incontinence Among Young Nulligravid Women: A Cross-Sectional Study
Source: Womens Health Rep (New Rochelle). 2025 May 12;6(1):546–55. doi: 10.1089/whr.2025.0004 (PMC12177332; doi:10.1089/whr.2025.0004)
Supplement: Supplementary Data S2 [file whr.2025.0004_supplementary_data_s2.pdf]

جامعة الشارقة
UNIVERSITY OF SHARJAH

Determining the Risk of Urinary Incontinence among Young Nulligravid Women

Questionnaire

Demographics and Medical history

1. How old are you?

   ☐ 18-20 years ☐ 21-25 years

2. What is your education level?

   ☐ Never attended school
   ☐ Primary or Intermediate
   ☐ Secondary
   ☐ University or college
   ☐ Postgraduate degree

3. What is your current status?

   ☐ Student
   ☐ Housewife
   ☐ Employed

4. Are you …?

   ☐ Single
   ☐ Currently married
   ☐ Previously married

5. How would you describe your ethnic origin?

- ❑ White/Caucasian
- ❑ Asian
- ❑ Arab
- ❑ African
- ❑ Others

6. Have you ever been diagnosed with any of these medical conditions?

a. Urinary tract or bladder infections more than 3 times in a year:

□ Yes □ No □ I don't know

b. Depression: □ Yes □ No □ I don't know

c. lung disease/Asthma: □ Yes □ No □ I don't know

Assessment of risk factors

1. How tall are you?

Centimeters…………………… □ I don't know

2. How much do you currently weigh?

Kilograms……………………. □ I don't know

3. Do you exercise?

□ Yes □ No

If yes, how frequently?

□ Not regular
□ 1-3 times per week
□ More than 3 times per week

If yes, which type of exercise (tick what applies)?

□ Walking
□ Swimming
□ Weight lifting
□ Others, (please indicate): -----------------------------

4. Do you currently take diuretics or “water pills”?
□ Yes □ No

5. Do you smoke?
□ Yes □ No

6. Have you ever been lifting more than 20 pounds on a regular basis?
□ Yes □ No □ I don’t know

7. Do you drink more than one cup of tea, coffee, coke, or other drinks with caffeine daily?
□ Yes □ No
If yes, on average, how many cups per day do you consume? ------------------

8. Do you empty your bladder even if you don’t need to do that “just in case”?

❑ Never

❑ Rarely

❑ Sometimes

❑ Often/Always

9. Do you delay going to the toilet until your bladder is very full?

❑ Never

❑ Rarely

❑ Sometimes

❑ Often/Always

10. Do you generally try to avoid using public toilets?
□ Yes □ No

Bladder symptoms

1. How many times a day do you empty your bladder?

☐ 3—5 ☐ 6—8 ☐ >8

Do you find this bothersome?

☐ Not at all

☐ Slightly

☐ Moderately

☐ Greatly

2. Do you wake more than twice at night to empty your bladder?

☐ Yes ☐ No

If yes, how much does it bother you?

☐ Not at all

☐ Slightly

☐ Moderately

☐ Greatly

3. When you first feel the need to pass urine, can you hold for around 30 min?

☐ Yes ☐ No

If not, how much does it bother you?

☐ Not at all

☐ Slightly

☐ Moderately

☐ Greatly

4. Do you leak urine if you don’t make it to the toilet in time?

- [ ] Yes
- [ ] No

If yes, how much does it bother you?

- [ ] Not at all
- [ ] Slightly
- [ ] Moderately
- [ ] Greatly

5. Do you leak urine during physical activity, coughing, laughing, or sneezing?

- [ ] Yes
- [ ] No

If yes, how much does it bother you?

- [ ] Not at all
- [ ] Slightly
- [ ] Moderately
- [ ] Greatly

6. Do you need to strain to pass urine or feel the urine stream is weak?

- [ ] Yes
- [ ] No

If yes, how much does it bother you?

- [ ] Not at all
- [ ] Slightly
- [ ] Moderately
- [ ] Greatly

7. Do you feel that you haven’t completely emptied your bladder?

- [ ] Yes
- [ ] No

If yes, how much does it bother you?

- [ ] Not at all
- [ ] Slightly
- [ ] Moderately
- [ ] Greatly

8. Do you have pain when passing urine?

- [ ] Yes
- [ ] No

If yes, how much does it bother you?

- [ ] Not at all
- [ ] Slightly
- [ ] Moderately
- [ ] Greatly

If you have never had urinary leakage, the questionnaire ends here. Thank you for the participation.

If you have urinary leakage, please answer the rest of the questions

9. When you leak urine, is it…….?

- [ ] Drops
- [ ] Large amount

10. Do you need to wear daily pads as a precaution for urine leaks?

- [ ] Yes
- [ ] No

If yes, how many a day?

- [ ] 2-3
- [ ] 4-5
- [ ] More than 5

11. Do you wet the bed?

- [ ] Yes
- [ ] No

If yes, how much does it bother you?

❑ Not at all ❑ Slightly

5

❑ Moderately ❑ Greatly

12. Have you ever asked a doctor, nurse, or another health professional for help with urine leakage?

❑ Yes ❑ No

13. Have you had any surgeries or procedures to correct urine leakage?

❑ Yes ❑ No

6

Quality of Life Assessment

Has urine leakage affected your:

1. Work or study?

❑ Not at all

❑ Slightly

❑ Moderately

❑ Greatly

2. Ability to do household chores (cooking, cleaning, laundry)?

❑ Not at all

❑ Slightly

❑ Moderately

❑ Greatly

3. Physical recreation such as walking, swimming, or other exercises?

❑ Not at all

❑ Slightly

- ❑ Moderately
- ❑ Greatly

4. Ability to travel by car or bus more than 30 minutes from home?

- ❑ Not at all
- ❑ Slightly
- ❑ Moderately
- ❑ Greatly

5. Participation in social activities outside your home?

- ❑ Not at all
- ❑ Slightly
- ❑ Moderately
- ❑ Greatly

6. Emotional health (nervousness, depression, etc.)?

- ❑ Not at all
- ❑ Slightly
- ❑ Moderately
- ❑ Greatly

7. Feeling frustrated?

- ❑ Not at all
- ❑ Slightly
- ❑ Moderately
- ❑ Greatly

Thank you for completing this questionnaire
