# Supplementary material for: Investigating the Risk Indicators of Urinary Incontinence Among Young Nulligravid Women: A Cross-Sectional Study
Source: Womens Health Rep (New Rochelle). 2025 May 12;6(1):546–55. doi: 10.1089/whr.2025.0004 (PMC12177332; doi:10.1089/whr.2025.0004)
Supplement: Supplementary Data S3 [file whr.2025.0004_supplementary_data_s3.docx]

Supplemental figure legend:

Numbers and Percentages of the level of bothersomeness from LUTS and UI among the study participants
